# Nutrition Habits and Oral Conditions Within a Representative Swiss Cohort of Adult Subjects

**DOI:** 10.3290/j.ohpd.c_2764

**Published:** 2026-08-26

**Authors:** Maurus Kurt Jaeggi, Christian Tennert, Maria Prasinou, Martin Schimmel, Andrea Roccuzzo, Guglielmo Campus, Roberta Borg-Bartolo

**Affiliations:** a Maurus Kurt Jaeggi Senior Dentist, Department of Restorative, Preventive and Paediatric Dentistry, School of Dental Medicine, University of Bern, Freiburgstrasse 7, CH-3010 Bern, Switzerland. Data collection, wrote original draft of the manuscript, proofread the manuscript.; b Christian Tennert Professor, Department of Restorative, Preventive and Paediatric Dentistry, School of Dental Medicine, University of Bern, Freiburgstrasse 7, CH-3010 Bern, Switzerland. Conceptualization, contributed substantially to discussion, proofread the manuscript.; c Maria Prasinou Dentist, Department of Restorative, Preventive and Paediatric Dentistry, School of Dental Medicine, University of Bern, Freiburgstrasse 7, CH-3010 Bern, Switzerland. Data collection, proofread the manuscript.; d Martin Schimmel Professor, Department of Reconstructive Dentistry and Gerodontology, School of Dental Medicine, University of Bern, Bern, Switzerland. Conceptualisation, performed statistical analysis, contributed substantially to discussion, analysed the results, proofread the manuscript.; e Andrea Roccuzzo Professor, Shanghai Perio-Implant Innovation Centre, Institute of Integrated Oral, Craniofacial and Sensory Research, Shanghai Ninth People’s Hospital, Shanghai Jiao Tong University School of Medicine, Shanghai, China; College of Stomatology, Shanghai Jiao Tong University, National Centre of Stomatology, National Clinical Research Centre for Oral Diseases, Shanghai Key Laboratory of Stomatology, Shanghai, China; Department of Periodontology, School of Dental Medicine, University of Bern, Bern, Switzerland. Data collection, contributed substantially to discussion, proofread the manuscript.; f Guglielmo Campus Professor, Department of Restorative, Preventive and Paediatric Dentistry, School of Dental Medicine, University of Bern, Freiburgstrasse 7, CH-3010 Bern, Switzerland; Department of Cariology, Institute of Odontology, Sahlgrenska Academy, University of Gothenburg, Gothenburg, Sweden; Department of Cariology, Saveetha Dental College and Hospitals, SIMATS, Chennai, 600077, India. Conceptualisation, performed statistical analysis, contributed substantially to discussion, analysed the results, proofread the manuscript.; g Roberta Borg-Bartolo Researcher, Department of Restorative, Preventive and Paediatric Dentistry, School of Dental Medicine, University of Bern, Freiburgstrasse 7, CH-3010 Bern, Switzerland; Data collection, performed statistical analysis, analysed the results, proofread the manuscript.

**Keywords:** caries, fibre, nutrition, oral health, periodontitis

## Abstract

**Purpose:**

This observational, cross-sectional study investigated the association between nutrition and oral health status in adults aged ≥ 45 years in the Canton of Bern, Switzerland.

**Methods and Materials:**

Citizens throughout the Canton of Bern, Switzerland, chosen by random sampling, were included in the study according to predefined inclusion and exclusion criteria. Participants filled out questionnaires covering their nutritional aspects and oral hygiene habits before undergoing clinical examination, including the assessment of oral hygiene, dental caries, periodontal disease, as well as prosthetic rehabilitation. Chi-square/Fisher tests, as well as binary and multinomial logistic regression, were performed to investigate potential associations.

**Results:**

275 participants (mean age 69.7 years; female 44%; male 56%) were included in the study. An association between higher age and poorer oral hygiene (RR = 1.07, 95% CI [1.024, 1.117]), higher caries experience (DMFT > 15: RR = 1.152, 95% CI [1.1, 1.207]), and higher prevalence of periodontal disease was observed (OR = 1.038, 95% CI [1.013, 1.063]). Participants who ate vegetables daily had a lower risk of having dental caries (DMFT > 15: RR = 0.437, 95% CI [0.237, 0.806]) and/or poor oral hygiene (RR = 0.309, 95% CI [0.141, 0.674]). Participants who ate nuts regularly presented with better oral hygiene than those who did not eat nuts (RR = 0.356, 95% CI [0.134–0.950]).

**Conclusion:**

Within its limitations, the present study demonstrated the importance of a healthy diet containing fibre-rich foods for the achievement and maintenance of good oral health.

In the majority of industrialised countries, including Switzerland, a significant rise in life expectancy with consequent demographic changes could be observed in the last century.^15,54,59^ This, together with the fact that a higher age is reached in a better state of health,^24^ results in a clear increase in healthcare demand. Therefore, it is crucial to focus on the improvement of strategies regarding the prevention and delay of negative effects of ageing, as well as the decrease in physiological function and health.^30^ A special area of interest in this topic is found in oral health and orofacial function, as the stomatognathic system has a critical role in every stage of an individual’s life. Maintaining good oral health and functional dentition are key factors in preserving quality of life and well-being in ageing.^3,10,14,18,52^ Impaired oral health may lead to pain,^53^ inflammation,^47^ and impaired function, especially reduced chewing ability,^36,49^ which can lead to insufficient nutritional intake and therefore, in the long term, to general health problems.^9,20,26,34^ The most prevalent pathologic oral health conditions in adults remain caries and periodontal disease.^29^ Both diseases represent a frequent problem, as they can lead to tooth loss if left untreated.^7,41^ An improvement in oral health conditions of adults over the last decades was observed, as previous epidemiological studies of other countries have shown.^22,38^ Adults (aged 35–44) in Germany show a reduction of the Decayed, Missing and Filled teeth index (DMFT index) from 16.1 (DMS III, 1997) to 8.3 (DMS VI, 2023)^21^. The same goes for adults (aged 35–49) in the United States, where a reduction of the DMFT Index from 11.3 (1999–2004) to 9.4 (2011–2016)^37^ has been reported. A decreasing trend for severe periodontal disease (Stage IV, CDC/AAP-Classification) in Germany was found.^40^ While in 1997 (DMS III) 18% of adults aged 35–44 suffered from severe periodontal disease, only 3.9% were found to have severe periodontal disease in 2023 (DMS VI).^12,33^ Nevertheless, both conditions remain a challenge for oral healthcare, as adults tend to have more natural teeth until old age.^22,38,50^

Caries and periodontitis are multifactorial diseases regarding their aetiology and progression.^32,56^ One of the main interacting factors besides oral hygiene and the host itself is nutrition. Nutrition and oral health have a well-established and multifaceted relationship. The development, progression and prevention of multiple oral diseases, including dental caries, periodontal disease and dental erosion, are strongly linked to nutritional habits of individuals.^16^ Frequent consumption of fermentable carbohydrates, especially sugars, is strongly associated with an increased proliferation of acidogenic/aciduric bacteria as well as the development and maintenance of an acidic environment, which, in case of frequent consumption, may lead to demineralisation of the dental hard tissues, resulting in dental caries.^58^ In the same way, an inadequate intake of essential nutrients like minerals, vitamins or micronutrients can impair the individual’s immune response and increase the susceptibility to periodontal disease.^31^ Previous studies have identified nutrients to have pro- or anti-inflammatory character, and a diet high in nutrients with anti-inflammatory character and low in nutrients with pro-inflammatory character has been found to reduce inflammation of the gingiva and/or the periodontium.^60,61,62^ Frequent consumption of processed foods, eg, foods rich in sugar, white flour, fast food, and limited awareness of their impact on oral health, has been found to contribute to the growing burden of oral diseases.^48^ These conditions affect oral health, function and aesthetics and are strongly associated with systemic health issues, such as cardiovascular disease or metabolic diseases like diabetes.^27,45^

Therefore, understanding the dietary determinants of oral health is crucial for the development of effective prevention strategies and public health policies, both globally and within specific populations such as in Switzerland. The aim of this study was to investigate the association between nutrition and oral health status, including oral hygiene, periodontal disease and dental caries in adults ≥ 45 years or older in the Canton of Bern, Switzerland. The hypothesis is that participants consuming a high amount of anti-inflammatory foods (vegetables, fruits, nuts and seeds) and low intake of sugar, processed foods and beverages have low levels of caries, gingival and periodontal parameters.

## METHODS AND MATERIALS

### Study Design

The study was designed as an observational, cross-sectional study, with a target population of subjects aged ≥ 45 years, living in the Canton of Bern, Switzerland. This paper is part of a larger research project to provide data on oral health, masticatory function, oral hygiene, nutritional aspects and oral health-related quality of life (OHRQoL) in adult/senior people living in the Canton of Bern, Switzerland.^43^ The study protocol received approval from the Ethical Committee of the Canton of Bern (KEK), Switzerland (Nos. 2020–02760 and 2021–01947). The examination was conducted according to the revised principles of the Helsinki Declaration (2013). Each participant received and signed an informed consent form before participating in the study.

#### Recruitment of the subjects

Contact information of the citizens of the Canton of Bern aged ≥ 45 or older was provided by the citizen centres of the different municipalities throughout the canton of Bern. The participants were chosen by random sampling according to a previously established methodology^6^. Before enrolment, the citizens of the Canton of Bern received information about the study and an invitation to participate via mail. The inclusion and exclusion criteria were as follows:

**Inclusion criteria:**

Residence in the Canton of Bern
Written declaration of informed consent
Age ≥ 45 years
Capacity to understand questionnaire items

**Exclusion criteria:**

Known allergic reaction to oral hygiene products and/or medication and/or dental material previously used in the mouth or pharynx.
Pathological changes of the oral mucosa, eg, acute ulcerative gingivitis, acute herpetic gingivostomatitis, recurrent aphthous ulceration or systemic illnesses with oral manifestations.
Antibiotic therapy within the past 3 weeks.

The enrolment process was performed as described in a previous publication of our research group.^43^ Questionnaires regarding oral hygiene, nutritional aspects, oral health, socioeconomic status, general health and lifestyle behaviours were sent out to the participants. An appointment for the oral examination was scheduled at the patient’s accommodation/residence and performed by two trained and previously calibrated examiners. At the appointment, the questionnaires were collected and checked for completeness. Any ambiguities or concerns about the questionnaires were discussed. No financial compensation or payment was provided to the participants.

#### Clinical examination

The oral examination included: oral hygiene (Approximal Plaque Index [API]),^25^ modified Papilla Bleeding Index (mPBI),^25,46^ dental caries assessment (ICDAS),^19^ DMFT Index,^4,5^ and periodontal disease assessment (Periodontal Screening Index [PSI]),^2,11^ as well as the record of present prosthetic rehabilitation (fixed or removable) or implants. Additionally, the masticatory performance was evaluated with a two-coloured chewing gum as reported previously.^44,57^ Besides the clinical examination, validated questionnaires regarding socioeconomic status, medical history, oral health behaviour, dietary assessment, and OHRQoL using the General Oral Health Assessment Index (GOHAI) were filled out. Questionnaires were available in German, French, and Italian.

#### Dietary assessment

Dietary assessment was performed using a questionnaire. This questionnaire is based on the DEGS1 questionnaire (Food Frequency Questionnaire) from the Robert Koch Institute.^17^ The DEGS1 questionnaire records the frequency of consumption and usual portion sizes of different food groups consumed in the past 4 weeks. Excerpts from this questionnaire were used that focus specifically on pro- and anti-inflammatory foods and ask about the frequency of consumption in the past 4 weeks. Participants answered questions about the type of food consumed, quantity and frequency of consumption. The questionnaire contained the following food groups:

Bread
Carbohydrates (including bread, potatoes, rice, pasta)
Sugar intake (including sweets, chocolate, ice cream, pastry)
Beverages, liquid foods
Animal products (including meat, fish, eggs, dairy)
Vegetables
Fruits
Legumes
Nuts and seeds

Food frequency was recorded by providing options such as never, rarely, once a day or once a week, several times a day or several times a week, or daily, depending on the specific food or food group based on the DEGS1 questionnaire.

### Calibration

The examiners were trained and calibrated before commencement of the study as reported in a previous publication.^6^ Briefly, intra- and inter-rater reliability were calculated using Cohen’s kappa scores and intraclass correlation coefficients (ICC) using the two-way mixed effects model, respectively. Kappa scores of 80–100% (*p* < 0.05, caries lesion calibration) and 100% (*p* < 0.05, dental restoration calibration) were obtained. The average ICCs for inter-rater reliability were 0.97 (95%CI = 0.94 – 0.98, *p* < 0.05) (caries, first calibration session), 0.95 (95%CI = 0.90–9.98, *p* < 0.05) (caries, second calibration), and 0.98 (95% CI = 0.97–0.99, *p* < 0.05) (dental restoration, first and second calibration).

#### Statistical analysis

The means and standard deviations (SD) describe the continuous variables, and the number of participants (n) and frequency (%) represent the categorical variables. Chi-square/Fisher tests were performed to assess the association between the four outcome variables, ie, API and mPBI (oral hygiene; API: low API = 0–25%, moderate API = 26–75%, high API > 75%, mPBI: low mPBI = 0–25%, moderate mPBI = 26–75%, high mPBI > 75%), PSI (periodontal disease; PSI scores 0–2 = no periodontitis, PSI score 3–4 = periodontitis present) and DMFT (caries experience, Category 1: DMFT 0–5, Category 2: DMFT 6–10, Category 3: DMFT 11–15, Category 4: DMFT > 15), and the independent variables. Furthermore, backward regression was carried out, where the initial model contained all the relevant predictors, followed by stepwise elimination of variables that least contributed to the model’s predictive accuracy. Multinomial regression was performed to assess the association between the outcome variables API, PBI and DMFT and the independent variables, and the adjusted relative risk ratios (RRR) were reported. Binary logistic regression was carried out to assess the association between periodontal disease and the independent variables. Adjusted odds ratios (OR) were reported. Statistical analysis was carried out using Stata SE18® (StataCorp, College Station, TX, USA) with statistical significance set at *p* < 0.05.

## RESULTS

**Fig 1a to d:**
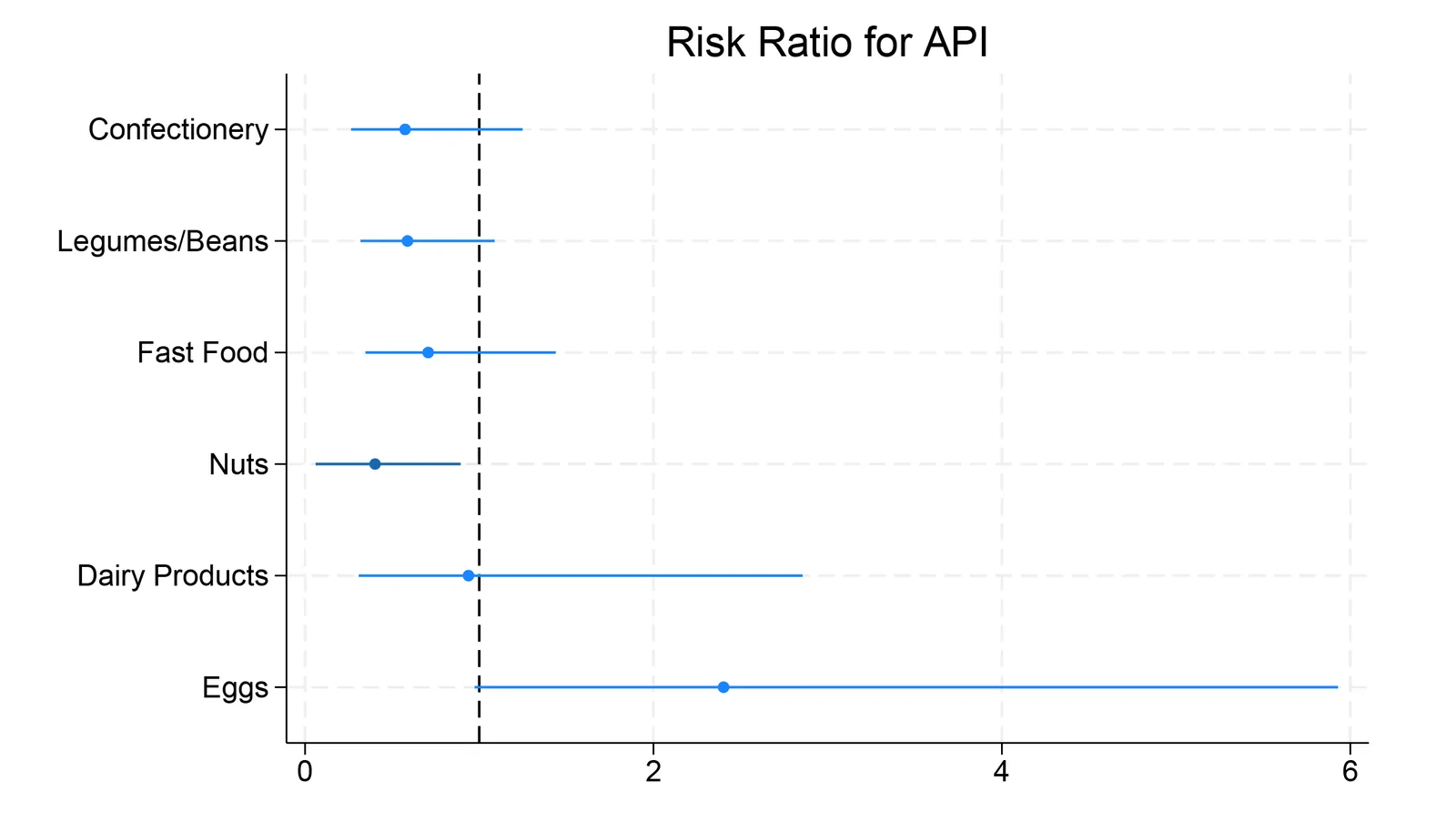

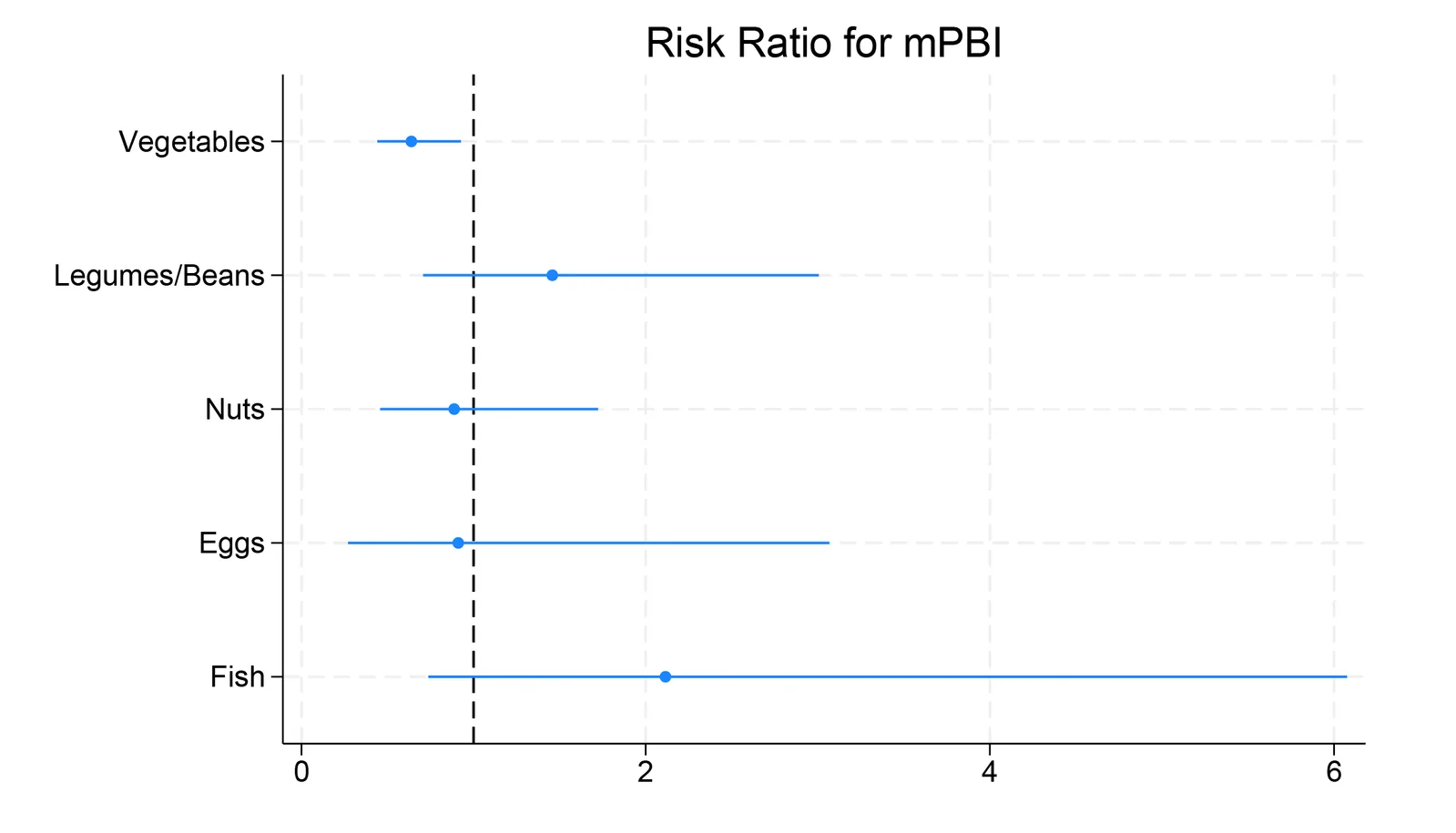

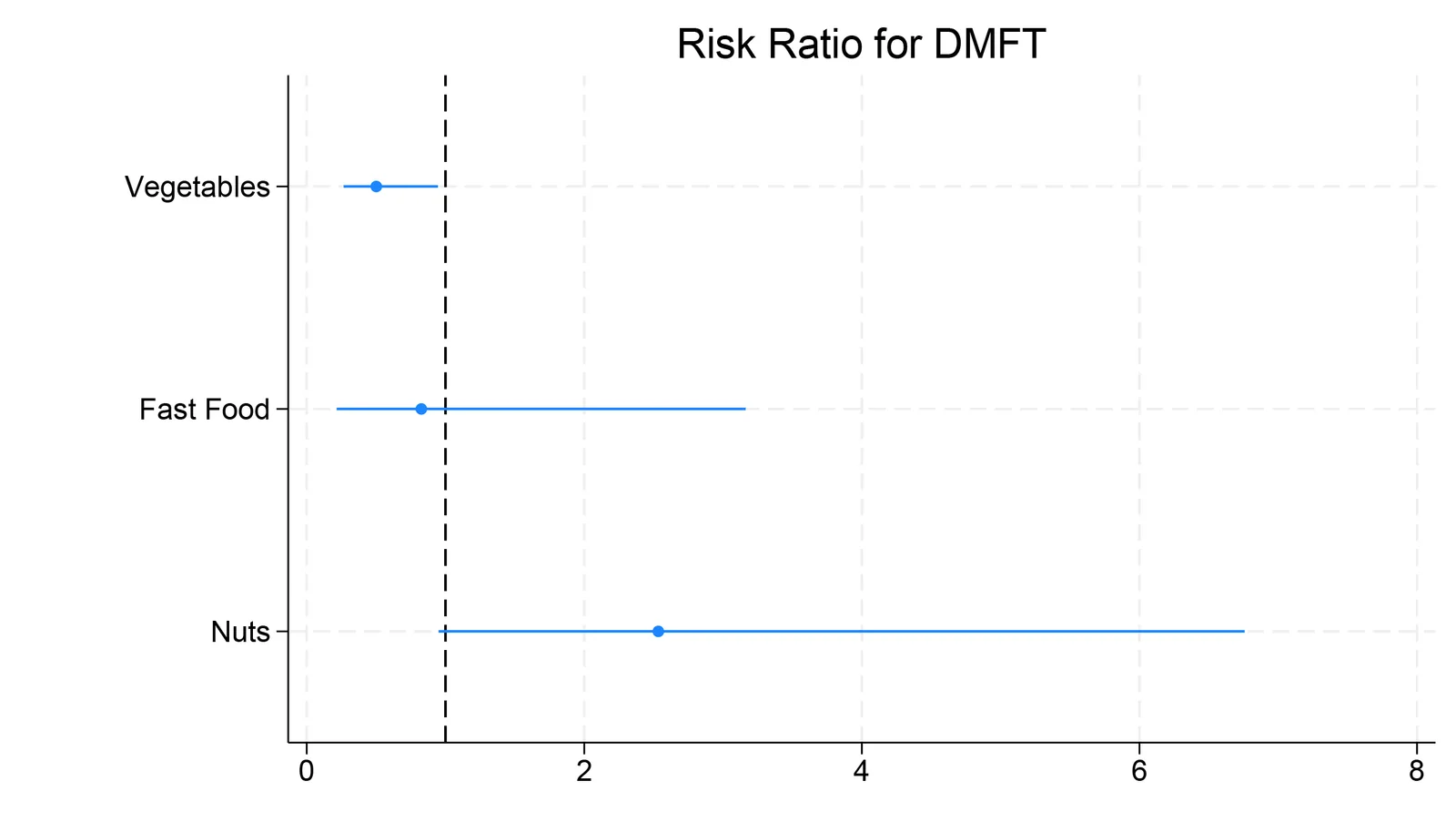

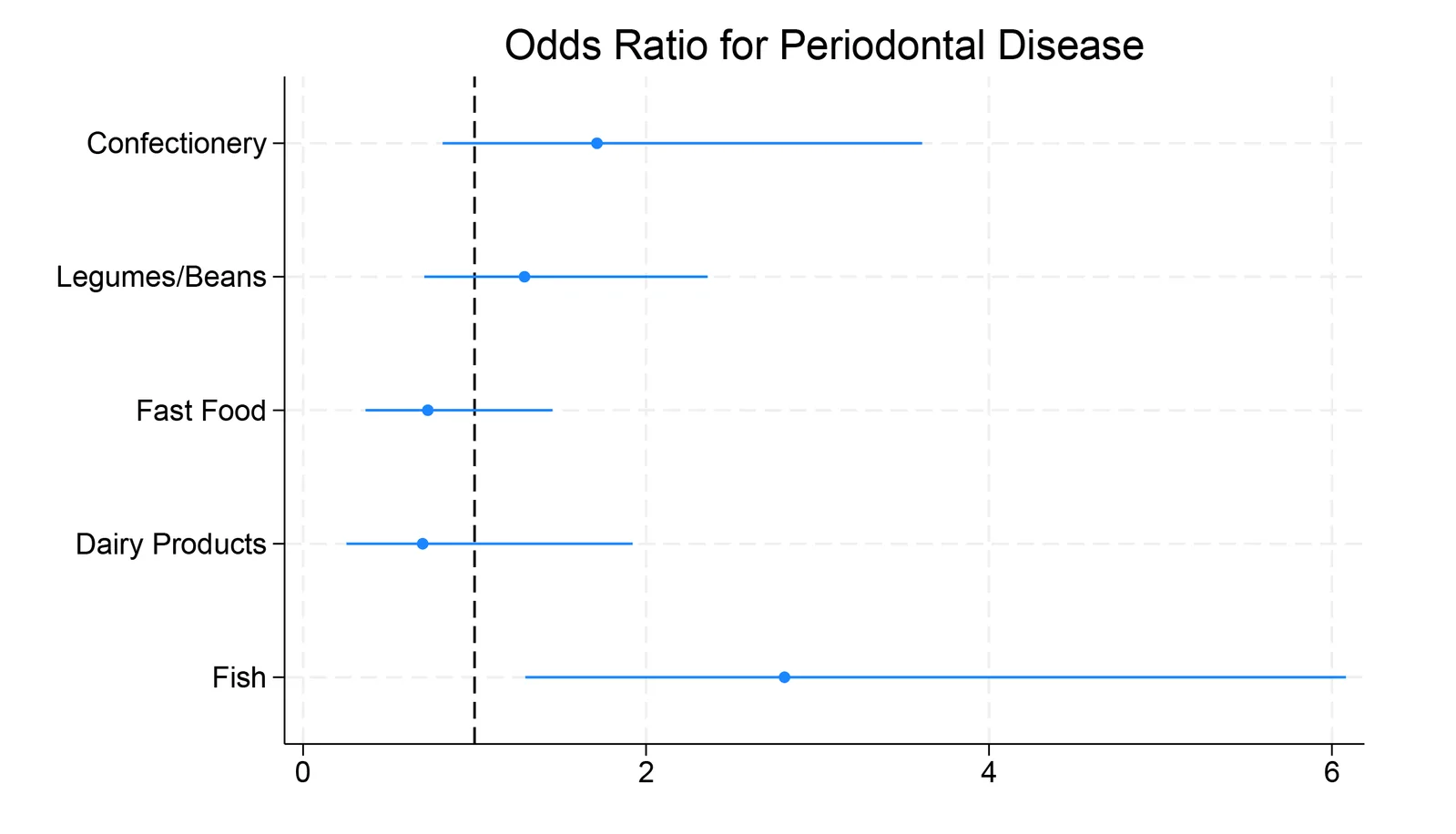
(a) Risk ratio for API; (b) risk ratio for mPBI; (c) risk ratio for DMFT; (d) odds ratio for periodontal disease.

275 participants were included in the study (154 (56%) males and 121 (44%) females, mean age 69.7 years (SD 11.6) and with a range of 45–99 years, response rate 8.4%). 90 (73%) lived in rural areas, and 139 (53%) had a tertiary level of education. The most prevalent medical condition was high blood pressure (33%), 15% had cardiovascular disease, 12% had rheumatoid arthritis, and 10% were diabetic. 7% of the participants were smokers. The majority brushed their teeth at least twice daily (221/86%) and visited the dentist regularly (n = 196, 79%). For the final statistical analysis, the following number of participants were included for each outcome variable: oral hygiene (API, mPBI): 270 (5 participants were either fully edentulous or had only one tooth present), periodontal disease: 262 (13 did not undergo a periodontal examination), dental caries experience: 275. In general, of the 275 participants 250 (91%) ate meat, 222 (81%) ate fish, 250 (91%) ate other animal products (milk, eggs, yogurt, cheese), 178 (65%) ate 2 or more portions of fruit per day, 236 (86%) ate 2 or more portions of vegetables per day, and 206 (80%) consume fast food two or more times a week (Table 1). χ^2^-test and/or Fisher’s exact test found a statistically significant (*p* < 0.05) association between API score and age (*p* = 0.007) and the consumption of nuts (*p* = 0.026). A statistically significant association could also be found between the mPBI-score and eating vegetables (*p* < 0.01), between the presence of periodontal disease and age (*p* < 0.01), gender (male/female) (*p* = 0.044) and eating fish (*p* = 0.027). A statistically significant association between DMFT-score and age (*p* < 0.01) and gender (male/female) (*p* = 0.028) was found. The multinomial logistic regression showed that older participants had a higher risk (RRR = 1.07, 95% CI 1.02–1.12) of having a high API score (> 75%), while those who ate nuts had a lower risk (RRR = 0.36, 95% CI 0.13–0.95) for high approximal plaque index scores (Fig 1a). Participants who ate vegetables on a daily basis had a lower risk (RRR = 0.31, 95% CI 0.14–0.67) of having high mPBI scores (> 75%) (Fig 1b). Older participants had a higher risk of having DMFT scores higher than 11 (11–15: RRR = 1.07, 95% CI 1.03–1.12 and DMFT > 15: RRR = 1.15, 95% CI 1.1–1.21]). On the other hand, eating vegetables was found to be negatively associated with DMFT (DMFT 6–10: RRR = 0.50, 95% CI 0.27–0.95 and DMFT > 15: RRR = 0.44, 95% CI 0.24, 0.81) (Fig 1c). The binary logistic regression showed that age (OR = 1.04, 95% CI 1.01–1.06) and eating fish (OR = 2.81, 95% CI 1.3–6.08) were associated with higher odds for having periodontal disease (Fig 1d, Table 2).

**Table 1:** Participant characteristics

Variable	Category	Participants n (%)
Age	45–64 years	78 (28.0)
	65–74 years	101 (36.7)
	> 75 years	96 (34.9)
Sex	male	154 (56.0)
	female	121 (44.0)
Education level	no tertiary education	129 (46.91)
	tertiary education	146 (53.09)
Employment level	retired	176 (64.0)
	in employment	99 (36.0)
Eating meat	do not eat meat	25 (9.09)
	eat meat	250 (90.91)
Eating fish	do not eat fish	53 (19.27)
	eat fish	222 (80.73)
2 or more portions of fruit per day	no	96 (35.04)
	yes	178 (64.96)
Eating vegetables	never/rarely	38 (13.87)
	several times per week	147 (53.65)
	daily	89 (32.48)
Eating salad	do not eat salad	71 (25.91)
	eat salad	203 (74.09)
Eating fast food	do not eat fast food	53 (20.46)
	eat fast food	206 (79.54)
Eating nuts	do not eat nuts	109 (39.64)
	eat nuts	166 (60.36)
Eating dairy products	do not eat dairy products	25 (9.09)
	eat dairy products	250 (90.91)
Eating eggs	do not eat eggs	34 (12.36)
	eat eggs	241 (87.64)
Periodontal disease	No perio disease	154 (58.78)
	perio disease	108 (41.22)
DMFT-score	DMFT < 5	37 (13.45)
	DMFT 6–10	54 (19.64)
	DMFT 11–15	97 (35.27)
	DMFT > 15	87 (31.64)
API	0–25%	110 (40.74)
	26–75%	133 (49.26)
	> 75%	27 (10.0)
mPBI	0–25%	203 (75.0)
	26–75%	55 (20.45)
	> 75%	11 (4.09)
		

**Table 2:** Regression analyses to assess the association between nutrition and oral health outcomes; API, mPBI, DMFT index, periodontal disease

Multinomial logistic regression				
Variable				
Binary logistic regression				
Variable				
**API**	0–25%	26–75%	> 75%	
age	1	**1.03 (1.01–1.06)**	**1.07 (1.02–1.12)**	
eating nuts	1	1.01 (0.56–1.80)	0.36 **(0.13–0.95)**	
			
**mPBI**	0–25%	26–75%	> 75%	
vegetables daily	1	**0.65 (0.45–0.93)**	**0.31 (0.14–0.67)**	
			
**DMFT score**	< 5	6–10	11–15	> 15
age	1	1.04 (1.0–1.09)	1.07 (1.03–1.12)	**1.15 (1.1–1.2)**
vegetables daily	1	**0.5 (0.27–0.95)**	0.63 (0.35–1.1)	**0.44 (0.24–0.81)**
**Periodontal disease**	no	yes		
age	1	**1.04 (1.01–1.06)**		
eating fish	1	**2.81 (1.3–6–08)**		


## DISCUSSION

The present study analysed the association between nutrition and oral health in subjects aged at least 45 years of age and residing within the Canton of Bern. Based on the obtained results, it can be concluded that older participants had a higher risk of having high API scores, DMFT scores above 6 and having periodontal disease. Regarding nutrition, participants who ate nuts regularly had a lower risk for high API scores (< 75%), while those who ate vegetables daily had a lower risk of having high mPBI (< 75%) or high DMFT scores (above 6), respectively. Evidence suggests that people who eat fruit and vegetables regularly have a higher number of teeth present.^23,28^ Although this was not analysed in the present study, a better oral health status, ie, lower DMFT scores and lower risk of having bleeding gums, was reported. Nuts and vegetables are low in sugars and high in fibre. Previous studies on the potential for inflammation of nutrients found that vegetables and nuts have anti-inflammatory effects.^51,62^

An interesting finding is that participants who reported eating fish had higher odds of having periodontal disease. There is currently inconclusive evidence in the literature regarding the effects of eating fish and periodontal disease. A study by Taber et al reported an association between several periodontal conditions (eg, increased bleeding on probing (BOP) and/or probing depth (PD)) and nutritional factors.^55^ On the other hand, a study by Ottosson et al found that 3-carboxy-4-methyl-5-propyl-2-furanpropionic acid (CMPF), a fatty fish marker, was associated with less periodontal inflammation and periodontitis.^39^ The type of fish is also important, because the content of anti-inflammatory omega-3-fatty acids seems the most important factor regarding consumption of fish and periodontitis.^60,61,62^ The type and origin of fish weren’t assessed, since it is difficult for the participants to give sufficient information. Wild-caught sea fish has a much higher content of omega-3-fatty acids compared to farmed or freshwater fish. Additionally, comparisons are difficult to make due to the differences in the study design, the low number of participants, as well as the applied case definition for periodontal health/disease. Further research is required regarding the association between fish consumption and periodontal disease.

As in the present study, several previous studies have shown similar associations between DMFT value and age, with an upward trend in mean DMFT value with increasing age.^13,29,42^ Although an association between nutrition and oral health has long been established, information on the worldwide situation on these factors is limited.^35^ Therefore, the collected data offer a unique value for the proxy representation of nutrition and oral health in a group of community dwellers in industrialised countries and for the further development of epidemiologic strategies on this topic. In the present study, factors commonly associated with periodontal disease and dental caries, such as diabetes and smoking, were not found to be associated with these conditions.^6,8^ This could be due to the low prevalence of these conditions among the participants of the study.

This study presents multiple strengths in its methodological design: the multilayered randomisation process enabled a precise identification of the target population while ensuring good representation across all ten regions of the canton of Bern; the implication of data gathered through a clinical examination together with the collection of data through questionnaires ensured a thorough data collection on oral health, nutrition, caries and periodontal disease; the questionnaire regarding nutrition portrays a broad expanse, which covers multiple aspects of nutrition of the past 4 weeks and does not only focus on sugar intake. In this way, not only caries is addressed, but also gingivitis and periodontitis. Limitations of the study include the low response rate of participants, which, however, is comparable to similar studies of this topic in Switzerland^1^. The results should be interpreted with caution, as the low response rate may introduce nonresponse and voluntary response bias. Another limitation is the cross-sectional design, which does not allow causal interpretation of results.^1^

Clinical implications of the current findings include the importance of in-depth instruction of the patients in the association of their daily dietary behaviour and oral health as well as the importance of an individualised approach and guidance in nutritional control. Furthermore, the study highlights the importance of an interprofessional approach including doctors, nutritionists and professionals who are more often in contact with elderly persons than dentists. Regarding the epidemiological aspects on national and international levels, the current findings highlight the importance of standardised, nationwide data collection approaches for optimised planning and execution of oral health and nutrition programmes. A more generalised approach would improve the comparability of the impact on oral health and dietary policies between different countries.

## CONCLUSION

Within its limitations, the present study identified the importance of a healthy diet, especially the regular consumption of vegetables and nuts, as a contributing factor in lower levels of gingival inflammation, DMFT scores and interdental plaque scores.

### Acknowledgements

The study was funded by the Lutz-Zürrer Stiftung, the Foundation Nakao for Worldwide Oral Health, Luzern, Switzerland, the Swiss Society for Periodontology and the Sana Foundation. The authors would like to thank the undergraduate students, Céline Gübelin, Clara Soliva, Sarah Dellenbach, Laura Kohli, Valérie Gasser, Lindsay Duay, James Imhof and Patric Maibach from the School of Dental Medicine, University of Bern, for their valuable help with the clinical examination process. The authors thank Ines Badertscher and Bernadette Rawyler for assisting with the design of the figures for this study. The authors also thank Beatrice Gerber and Monica Fuhrer, Department of Reconstructive Dentistry and Gerodontology, School of Dental Medicine University of Bern, Freiburgstrasse 7, 3010 Bern, Switzerland for collecting the chewing gum samples and taking photographs of the samples and Flavio Gerber for evaluating the photographs of the samples.
